# Progress Toward Poliomyelitis Eradication — Afghanistan, January 2018–May 2019

**DOI:** 10.15585/mmwr.mm6833a4

**Published:** 2019-08-23

**Authors:** Maureen Martinez, Hemant Shukla, Joanna Nikulin, Chukwuma Mbaeyi, Jaume Jorba, Derek Ehrhardt

**Affiliations:** ^1^Global Immunization Division, Center for Global Health, CDC; ^2^Polio Eradication Department, World Health Organization, Geneva, Switzerland; ^3^Division of Viral Diseases, National Center for Immunization and Respiratory Diseases, CDC.

Since October 2016, Afghanistan and Pakistan have been the only countries with reported cases of wild poliovirus type 1 (WPV1) (*1*). In Afghanistan, although the number of cases had declined during 2013–2016, the polio eradication program experienced challenges during 2017–2019. This report describes polio eradication activities and progress in Afghanistan during January 2018–May 2019 and updates previous reports (*2*,*3*). During May–December 2018, insurgent groups (antigovernment elements) banned house-to-house vaccination in most southern and southeastern provinces, leaving approximately 1 million children inaccessible to oral poliovirus vaccine (OPV) administration. During January–April 2019, vaccination targeting children at designated community sites (site-to-site vaccination) was permitted; however, at the end of April 2019, vaccination campaigns were banned nationally. During 2018, a total of 21 WPV1 cases were reported in Afghanistan, compared with 14 during 2017. During January–May 2019, 10 WPV1 cases were reported (as of May 31), compared with eight during January–May 2018. Sewage sample–testing takes place at 20 sites in the highest-risk areas for poliovirus circulation; 17 have detected WPV1 since January 2017, primarily in the southern and eastern provinces. Continued discussion with antigovernment elements to resume house-to-house campaigns is important to achieving polio eradication in Afghanistan. To increase community support for vaccination, collaboration among humanitarian service agencies to address other urgent health and basic needs is critical.

## Immunization Activities

The World Health Organization (WHO) and UNICEF estimated that national routine vaccination coverage of children aged <12 months with the third dose of OPV (OPV3) in Afghanistan was 73% in both 2017 and 2018 (*4*). Routine immunization services were not generally available in the southern and eastern regions. In both 2017 and 2018, 68% of children aged 6–23 months with nonpolio acute flaccid paralysis (NPAFP) had a history of receipt of 3 OPV doses through routine immunization services, which is a proxy indicator of national OPV3 coverage. The proportion of children aged 6–23 months with NPAFP who never received OPV through routine immunization services or supplementary immunization activities (SIAs)* (i.e., zero-dose children) was 1% nationally in 2018; the largest percentages of these children were from the southern provinces of Kandahar (26%) and Helmand (15%). Coverage with injectable inactivated poliovirus vaccine (IPV), which was introduced into all OPV-using countries in 2016 in conjunction with the global, synchronized switch from trivalent OPV (containing vaccine virus types 1, 2, and 3) to bivalent OPV (bOPV, containing types 1 and 3), was estimated at 66% in 2018.

During January 2018–May 2019, SIAs targeted children aged <5 years for receipt of monovalent OPV (mOPV1, containing only type 1) or bOPV, including 2 national immunization days (NIDs), 5 subnational immunization days, three responses to WPV1-positive cases, five mop-up SIAs, and one short-interval additional dose campaign (SIAD).^†^ NIDs targeted 9,999,227 children aged <5 years. During SIAs, IPV was administered to 549,557 children aged 4–59 months who lived in very high-risk districts for WPV1 circulation or in areas that had been inaccessible during previous SIAs.

Children missed during SIAs are classified either as inaccessible because of campaign bans or accessible but missed because of campaign quality issues. During the March 2018 NID, according to postcampaign assessments, an estimated 110,591 (1.2%) targeted children were inaccessible for campaigns, and 339,474 (3.6%) were accessible but missed. During the August 2018 NID, which occurred during the May–August 2018 ban on SIAs in areas held by anti-government elements, a total of 1,324,132 (13.2%) targeted children were inaccessible, and 300,471 (3%) were accessible but missed.

The standard SIA approach for polio eradication involves house-to-house OPV vaccination. In November 2018 and January 2019, the polio program gained access for site-to-site campaigns in some areas of the southeastern and southern regions. During the April 2019 NID, the number of missed children among those targeted was reduced to 743,776 (7.4%), including 449,756 (4.5%) who were inaccessible and 294,020 (2.9%) who were accessible but missed because of campaign quality issues.

Lot quality assurance sampling^§^ surveys are used to assess the quality of SIAs in areas where postcampaign monitoring is permitted. Depending on the number of unvaccinated persons in the survey sample, districts were marked as either passed at 90% (estimated coverage ≥90%), passed at 80% (estimated coverage 80%–90%), or failed at <80% (estimated coverage <80%). At the 80% threshold, 8.3% of districts failed in the March 2018 NID, and 3% failed in the August 2018 NID. During the March 2019 NID, 30% of districts failed at the 80% threshold. In March 2019, the passing threshold was raised to 90%. The inability to conduct house-based vaccination campaign evaluations after designated site campaigns resulted in unreliable coverage estimates.

Children aged <5 years are also targeted for vaccination along major travel routes throughout the country, at transit points from inaccessible areas, and at border crossing points with Iran and Pakistan. During January 2018–April 2019, approximately 18,490,713 doses of OPV were administered at transit points and approximately 1,540,171 doses at border crossings.

## Poliovirus Surveillance

**Acute flaccid paralysis (AFP) surveillance**. Detection of ≥2 NPAFP cases per 100,000 persons aged <15 years is considered sufficiently sensitive surveillance to detect a case of polio; to assess quality of case investigation, 80% of AFP cases should have adequate stool specimens collected.^¶^ The polio surveillance network includes approximately 800 AFP focal points; 2,500 health facilities; and 35,000 reporting community volunteers. In 2018, the national NPAFP rate was 17 per 100,000 persons aged <15 years for areas across all SIA categories (accessible, inaccessible, and partially accessible) (regional range = 11–21) (Table). The percentage of AFP cases with adequate specimens was 94% (≥85% across all SIA access categories) (regional range = 87%–97%).

**TABLE:** Acute flaccid paralysis (AFP) surveillance indicators and reported cases of wild poliovirus (WPV), by region and period — Afghanistan, January 2018–May 2019*

Region of Afghanistan	AFP surveillance indicators (2018)			No. of WPV cases reported		
	No. of AFP cases	Nonpolio AFP rate^†^	% of AFP cases with adequate stool specimens^§^	Jan–May 2018	Jun–Dec 2018	Jan–May 2019
**All regions**	**3,357**	**17**	**94**	**8**	**13**	**10**
Badakhshan	68	11	96	0	0	0
Central	615	13	97	0	0	0
Eastern	400	20	94	3	3	1
Northeastern	436	19	94	0	0	0
Northern	355	14	93	0	0	0
Southeastern	299	15	96	0	0	0
Southern	592	17	87	5	10	9
Western	592	21	96	0	0	0

* Data current as of May 31, 2019.

^†^ Cases per 100,000 persons aged <15 years. Considering underlying rate of nonpolio AFP in population, threshold indicating adequate surveillance is ≥2 nonpolio AFP cases per 100,000 persons aged <15 years.

^§^ Surveillance target is that ≥80% of AFP cases have adequate stool specimens collected. Adequate stool specimens are defined as two stool specimens of sufficient quality for laboratory analysis, collected ≥24 hours apart, both within 14 days of paralysis onset, and arriving in good condition at a World Health Organization–accredited laboratory with reverse cold chain maintained, without leakage or desiccation, and with proper documentation.

**Environmental surveillance.** Supplementary poliovirus surveillance in Afghanistan is conducted monthly through sampling of sewage at 20 sites in nine provinces. WPV1 was detected in two of 184 (1%) specimens tested in 2016, 42 of 316 (13%) specimens tested in 2017, 83 of 336 (25%) specimens tested in 2018, and 25 of 128 specimens (23%) collected in 2019 (as of May 31); all detections of poliovirus in 2019 occurred at sites in Helmand, Kandahar (southern), and Nangarhar (eastern) provinces.

## Epidemiology of WPV Cases

**FIGURE 1:**
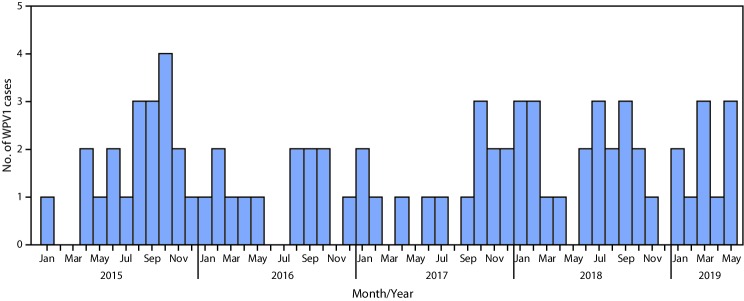
Number of wild poliovirus type 1 (WPV1) cases (N = 78), by month — Afghanistan, January 2015–May 2019

**FIGURE 2:**
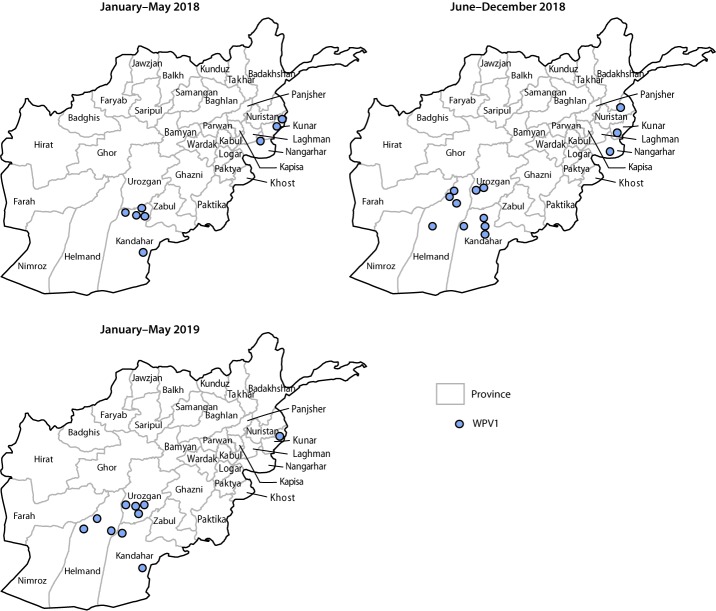
Cases of wild poliovirus type 1 (WPV1),* by province — Afghanistan, January 2018–May 2019 * Each dot represents one case. Location of dot on map does not represent actual location of case.

During 2018, 21 WPV1 cases were reported from 14 districts in six provinces (Helmand, Kandahar, Kunar, Nangarhar, Nuristan, and Urozgan), compared with 14 WPV1 cases reported from nine districts in five provinces (Helmand, Kandahar, Kunduz, Nangarhar, and Zabul) during 2017. During January–May 2019, 10 cases were reported from nine districts in four provinces (Helmand, Kandahar, Kunar, and Urozgan), compared with eight cases from five districts in three provinces (Kandahar, Kunar, and Nangarhar) during January–May 2018 (Figure 1) (Figure 2). Among the 31 cases reported during January 2018–May 2019, 20 (65%) were among children aged <36 months. Ten (32%) children had never received OPV through routine immunization or SIAs, three (10%) had received 1 or 2 doses, and 18 (58%) had received ≥3 doses each; 21 (68%) of the 31 children had never received OPV through routine immunization, but some had received OPV through SIAs.

Genomic sequence analysis of poliovirus isolates identified multiple episodes of cross-border transmission between Afghanistan and Pakistan during 2018–2019, with sustained local transmission in both countries. Seven (23%) of 31 isolates from patients with AFP and 13 (10%) of 111 isolates from environmental testing identified in Afghanistan had closest genetic links to earlier WPV1 isolates from Pakistan; the remaining WPV1 cases and isolates were most closely linked to cases and isolates from within Afghanistan. During January 2018–May 2019, two genetic clusters (viruses sharing ≥95% sequence identity) were detected among AFP cases. Transmission in the provinces of the eastern and southern regions is largely from independent genetic clusters. During January 2018–May 2019, four orphan viruses** were detected in environmental isolates from Helmand, Kabul, Kandahar (southern), and Nangarhar (eastern) provinces, signaling some AFP surveillance gaps.

## Discussion

Although the number of WPV1 cases has marginally increased in Afghanistan during 2017–2019 and circulation has remained confined to the southern and eastern regions of the country, the geographic extent of WPV1 circulation has increased at provincial and district levels in 2019. Although the Afghanistan program has succeeded in interrupting internal circulation in certain areas of the country in the past, internal WPV1 circulation has persisted since 2016.

When SIAs are conducted in accessible areas, a small but constant proportion of children continues to be missed because of suboptimal SIA planning, team performance issues, or both. Vaccine refusals and polio campaign fatigue continue in areas where populations without many basic services are still offered monthly polio vaccination. UNICEF has piloted water and sanitation projects in high-refusal areas, but the impact is unclear. Children reported absent during campaigns might represent undeclared caretaker refusals; further investigation might allow identification of underlying reasons that children are not present and help guide remedial action. Extending basic health and public services could improve community trust in such areas.

However, inaccessibility, compounded by the nationwide ban on vaccination campaigns, currently is the most substantial barrier to polio eradication in Afghanistan. Antigovernment elements in southern provinces have frequently banned house-to-house vaccination in the past, but over many periods, local access was permitted after discussions with local leaders. Antigovernment elements in the eastern provinces have imposed intermittent bans on house-to-house activities since 2016. To date, efforts to resume house-to-house campaigns after the nationwide ban have been unsuccessful; however, resumption of these campaigns is vital to achieving population immunity high enough to interrupt virus transmission, particularly in the southern and eastern provinces.

As long as the ban on vaccination campaigns continues, routine immunization services provide the most critical opportunity for polio vaccination in the country, but these services are extremely limited in many parts of the country. Enhanced efforts by national and international immunization partners can facilitate systematic provision of routine immunization activities through fixed, mobile, and outreach approaches, particularly in the most needed areas.

Solutions for improving immunization coverage and providing basic health services, including in areas held by antigovernment elements, are necessary to make substantial progress toward polio eradication in Afghanistan. These solutions will require close partnership from the highest levels of government and all international partners.

**Summary**

**What is already known about this topic?**

Wild poliovirus circulation continues in Afghanistan.

**What is added by this report?**

With bans on house-to-house vaccination campaigns in many provinces since May 2018 and a nationwide ban since April 2019, wild poliovirus circulation has increased during 2018–2019.

**What are the implications for public health practice?**

Routine immunization systems, which are critically weak in the provinces where wild poliovirus is currently circulating, are vital to polio eradication efforts, particularly until bans on campaigns are lifted. Successful discussions with local leaders have facilitated house-to-house campaigns in the past, and such campaigns are essential to interrupting wild poliovirus virus transmission.

## Footnotes

* SIAs are mass house-to-house campaigns targeting children aged <5 years with OPV, regardless of vaccination history.

† A mop-up SIA is a door-to-door immunization campaign carried out in specific areas where the virus is known or suspected to be still circulating. A SIAD follows another campaign by <2 weeks.

§ Lot quality assurance sampling is a rapid method used to assess the quality of vaccination activities after SIAs in predefined areas such as health districts (referred to as “lots”), using a small sample size. Lot quality assurance sampling involves dividing the population into lots and ascertaining receipt of vaccination by randomly selected persons within each lot. If the number of unvaccinated persons in the sample exceeds a predetermined value, then the lot is classified as having an unsatisfactory level of vaccination coverage, and mop-up activities are recommended. If the threshold of ≥90% is met (Afghanistan program guidelines have recently increased the threshold from ≥80%), the area or district is classified as having passed, although mop-up activities might still be indicated in certain areas.

¶ Surveillance target is that ≥80% of AFP cases have adequate stool specimens collected. Adequate stool specimens are defined as two stool specimens of sufficient quality for laboratory analysis, collected ≥24 hours apart, both within 14 days of paralysis onset, and arriving in good condition at a World Health Organization–accredited laboratory with reverse cold chain maintained, without leakage or desiccation, and with proper documentation.

** Orphan viruses are ≥1.5% divergent from their closest genetic match (i.e., ≤98.5% of a match).
